# Genotype–Phenotype Relationships in Azole-Resistant *Aspergillus*: Two Sides of the Same Coin

**DOI:** 10.3390/jof12040290

**Published:** 2026-04-18

**Authors:** Merlijn H. I. van Haren, Willem J. G. Melchers, Jianhua Zhang, Sarah Dellière, Christine C. Bii, Felicia A. Stanford, Michael Voetz, P. Lewis White, Paul S. Dyer, Suzan D. Pas, Paul E. Verweij, Jochem B. Buil

**Affiliations:** 1 Department of Medical Microbiology, Radboudumc Community for Infectious Diseases, Radboudumc, 6525 GA Nijmegen, The Netherlands; merlijn.vanharen@radboudumc.nl (M.H.I.v.H.); willem.melchers@radboudumc.nl (W.J.G.M.);; 2 Parasitology-Mycology Department, Hôpital Saint-Louis Paris, Assistance Publique-Hôpitaux de Paris, 75010 Paris, France; 3 Immunology of Fungal Infection, Institut Pasteur, Université de Paris Cité, 75015 Paris, France; 4 Mycology Division, Center for Microbiology Research, Kenya Medical Research Institute, Nairobi P.O. Box 54840 00200, Kenya; 5 School of Life Sciences, University of Nottingham, Nottingham NG7 2TQ, UKpaul.dyer@nottingham.ac.uk (P.S.D.); 6 Xebios Diagnostics GmbH, 40597 Düsseldorf, Germany; michael.voetz@xebios.com; 7 Mycology Reference Laboratory, Public Health Wales, Cardiff CF14 4XW, UK; 8 Radboudumc-CWZ Center of Expertise for Mycology (RCEM), Radboud University Medical Center, 6525 GA Nijmegen, The Netherlands

**Keywords:** *Aspergillus fumigatus*, triazole resistance, mutation management

## Abstract

*Aspergillus fumigatus* is a leading cause of invasive fungal disease in humans and is classified as a critical priority threat by the World Health Organization. Triazole antifungals remain the cornerstone of therapy, yet their effectiveness is steadily being eroded by the continuous rise in drug resistance. Most resistance mechanisms trace back to mutations in Cyp51A, spawning well-defined genotypes such as TR_34_/L98H and TR_46_/Y121F/T289A. However, the Cyp51A genotype–phenotype landscape in *A. fumigatus* is far from straightforward. Isolates that share an identical TR genotype can display strikingly divergent susceptibility profiles, and mutational hotspots in Cyp51A, such as G54, M220 and G448, are linked to varying resistances, challenging assumptions about predictable resistance behavior. Complicating matters further, an expanding array of resistance mechanisms, independent of Cyp51A, is now being uncovered. This review summarizes the current state of knowledge on azole resistance in *A. fumigatus*, dissecting the intricate genotype–phenotype relationships, spotlighting emerging non-Cyp51A pathways and outlining future strategies to enhance the detection and clinical management of antifungal resistance.

## 1. Introduction

Fungal infections represent a rapidly escalating global human health threat, imposing substantial morbidity, mortality and subsequent economic burdens [1]. Among these pathogens, certain *Aspergillus* species can cause aspergillosis, which ranges from allergic forms to invasive aspergillosis (IA), a life-threatening infection with mortality rates that remain high despite advances in clinical management. The urgency of this threat was underscored by the World Health Organization’s 2022 designation of *Aspergillus fumigatus* as a critical priority fungal pathogen, reflecting its ubiquity, virulence and rising drug resistance [2,3]. The species has a main ecological niche as a saprotroph, with the small airborne conidia of *A. fumigatus* widespread in air, meaning that they are frequently inhaled. In immunocompromised hosts, the species can act as an opportunistic pathogen due to the germination of conidia within the respiratory tract, where they evade early immune defenses. Once established, the fungus secretes potent secondary metabolites—most notably, gliotoxin—whose immunosuppressive and cytotoxic activity further sabotage host immunity [4]. In addition, azole resistance is observed in patients with chronic pulmonary aspergillosis (CPA), a condition with direct evidence of *Aspergillus* infection by microscopy or culture, or immunological response and the exclusion of alternative diagnoses all present for at least three months, which can require over twelve months of azole therapy [5]. In a study, azole resistance was observed in 13% of patients treated with itraconazole (ITC) and 5% of patients treated with voriconazole (VRC) [6]. As azoles represent the only antifungal class that can be given orally, resistance severely complicates patient management as long-term intravenous therapy is required in azole-resistant CPA. In combination with the severely limited antifungal armamentarium and the global rise in at-risk patient populations, these features create a confluence of events, making the need for new therapeutic strategies, as well as improved disease surveillance, increasingly urgent [7,8].

Triazole antifungals, including ITC, VRC, posaconazole (POS) and isavuconazole (ISA), remain the first-line therapy for IA because of their clinical efficacy, manageable toxicity and oral availability [9,10]. These agents target Cyp51A, the C-14α sterol demethylase essential for ergosterol biosynthesis, by binding its heme cofactor (Fe-protoporphyrin IX) [11]. This blockade induces toxic sterol intermediates, disrupts membrane integrity and ultimately halts fungal growth, with typical cidal activity against *Aspergillus* spp. [12,13,14]. However, the emergence and global spread of triazole resistance now jeopardize the long-term viability of this drug class. Resistance can arise during prolonged clinical therapy, but environmental selection has become a more prominent driver [15]. In patients with IA, resistance mutations associated with environmental resistance selection are almost exclusively found, while, in CPA, both environmental and in-host mutations can be observed [16,17,18,19]. The widespread use of agricultural azole fungicides has created intense and sustained selective pressure, enabling the evolution of resistant *Aspergillus* strains in the environment that readily disperse via airborne spores and can later infect humans [20,21,22]. Due to chemical similarity, resistance to azole fungicides leads to cross-resistance to medical azoles. Clinically, resistance correlates with poor diagnosis, treatment failure and excess mortality [23].

Most azole resistance mechanisms in *A. fumigatus* trace back to mutations in the *cyp51A* gene. The most impactful and globally dominant mechanisms are tandem repeat (TR) expansions in the promoter region, typically coupled with nonsynonymous mutations, such as TR_34_/L98H and TR_46_/Y121F/T289A, that drive *cyp51A* overexpression and reduce the azole-binding affinity [24]. These environmentally selected genotypes now constitute the majority of azole-resistant isolates worldwide, prompting the development of molecular detection assays [25,26,27,28,29]. Besides TR expansions, mutational hotspots in *cyp51A* related to azole resistance, such as G54, M220 and G448, are often associated with particular azole resistance profiles [30,31,32]. However, the presence of mutations in Cyp51A alone does not fully explain the phenotypic diversity observed in clinical isolates [33]. Additional resistance mechanisms, including the upregulation of efflux transporters, compensatory stress response pathways and complex regulatory network rewiring, introduce further layers of complexity. These factors make associations between genotypes and phenotypes regarding azole resistance difficult to predict for *A. fumigatus* and weaken the predictive power of single-gene diagnostics, highlighting the need for integrative approaches that combine genomics, transcriptomics and functional assays.

This review will evaluate current knowledge of genotype-to-phenotype relationships in azole-resistant *A. fumigatus*, looking at mutations in Cyp51A and other known mechanisms. Additionally, phenotypic and genotypic diagnostic methods used to determine antifungal drug resistance are examined, highlighting the challenges that hinder effective surveillance. Emerging strategies designed to detect, predict and ultimately mitigate triazole resistance are also evaluated.

## 2. Cyp51A Genotype–Phenotype Relation

Wildtype *A. fumigatus* is intrinsically resistant to fluconazole but susceptible to other mold-active triazoles, making VRC and ISA the preferred first-line agents for the treatment of IA [34,35]. As these drugs exert their activity through the inhibition of Cyp51A, nonsynonymous mutations in the *cyp51A* gene have emerged as the dominant mechanism of acquired resistance reported from clinical isolates. Cyp51A is a cytochrome P450 enzyme harboring a heme moiety within its active site; it catalyzes the oxidative demethylation of sterol precursors at the C-14 position—a critical step in ergosterol biosynthesis and consequent fungal membrane integrity. Azoles act as competitive Cyp51A inhibitors by coordinating with the iron of the heme moiety, thereby blocking sterol demethylation, promoting the accumulation of toxic intermediates and ultimately arresting fungal growth [36]. Cyp51B has around 60% identity with Cyp51A [37], and gene deletion experiments suggest that Cyp51A confers intrinsic resistance to fluconazole, while Cyp51B is susceptible to all triazoles [38].

Following the European Committee on Antimicrobial Susceptibility Testing (EUCAST) antifungal clinical breakpoints updated in June 2025, *A. fumigatus* is classified as resistant to ITC, VRC and ISA when the minimum inhibitory concentration (MIC) exceeds 1 µg/mL and is resistant to POS when the MIC exceeds 0.13 µg/mL (Table 1) [39]. The two most prevalent *cyp51A* genotypes conferring multi-azole resistance are TR_34_/L98H and TR_46_/Y121F/T289A. In a nationwide collection of 1979 *A. fumigatus* isolates resistant to at least one azole recovered in the Netherlands between 1994 and 2022, 1338 (67.6%) carried TR_34_/L98H and 332 (16.8%) harbored TR_46_/Y121F/T289A without additional *cyp51A* mutations, accounting for more than 84% of the azole resistance-associated genotypes [40]. TR_34_/L98H is most frequently associated with a pan-triazole-resistant phenotype, observed in 916 of 1338 isolates (68.5%). The TR_34_/L98H genotype has also been found in azole-resistant isolates from other clinical cohorts, such as 39/66 (59.0%) in Denmark [41], 123/143 (86.0%) in Germany [42] and 24/45 (53.3%) in Spain [43]. In contrast, TR_46_/Y121F/T289A, first reported in 2013, is characterized by exceptionally high VRC MICs, while susceptibility to ITC and POS may be retained in some isolates [44]. For both genotypes, resistance arises from the synergistic interaction between TR expansions and missense mutations, as either alteration alone results only in modest MIC elevations (Table 1). The TR duplication, located in the promotor sequence, drives up to eightfold overexpression of *cyp51A* [45], while the L98H substitution might induce conformational changes in the BC loop and helix C of Cyp51A that weaken azole binding without substantially compromising enzymatic activity [46]. Structural analyses of Cyp51A carrying the TR_46_/Y121F/T289A allele indicate that these mutations alter azole binding in a highly drug-specific manner. The Y121F substitution disrupts a water-mediated hydrogen bond network that normally stabilizes the interaction between the tertiary alcohol of voriconazole and the active site, markedly reducing VRC affinity. In contrast, itraconazole and posaconazole do not rely on this hydrogen bond network, and their binding is instead supported by hydrophobic interactions between their extended side chains and the substrate/ligand entry channel, a region that VRC cannot effectively engage. The T289A mutation further perturbs helix I, weakening VRC binding even further, while having a comparatively smaller effect on ITC and POS [47,48,49]. The net result is the overproduction of a structurally intact but poorly inhibited target enzyme, leading to clinically significant azole resistance.

Further amplifying this mechanism, a genotype containing three sequential copies of TR_46_ (TR_46_^3^) was identified in 2017 following the prolonged incubation of *A. fumigatus* in azole-treated compost [50]. This configuration conferred *cyp51A* expression levels exceeding those observed in wildtype, TR_34_ or TR_46_ isolates. Chromatin immunoprecipitation (ChIP) experiments demonstrated the direct binding of the transcription factors AtrR and SrbA to the TR-containing promotor region, mechanistically linking repeat expansion to the transcriptional upregulation of *cyp51A* [51,52].

There is a long-standing paradigm that tandem repeat mutations such as TR_34_/L98H and TR_46_/Y121F/T289A evolve as a result of the exposure of *A. fumigatus* reservoirs to azoles in the environment [22,29,53]. However, it is important to note that TR-based resistance mechanisms are not confined exclusively to environmental selection. A striking example is the recovery of a *cyp51A* TR_120_/F46Y/M172V/E427K isolate from a patient receiving prolonged azole therapy [54]. This strain exhibited a pan-azole-resistant phenotype (Table 1) and differed by no short tandem repeat (STR) markers and only 41 single-nucleotide polymorphisms (SNPs) from a previously isolated F46Y/M172V/E427K strain, strongly indicating that the TR_120_ expansion arose de novo under in-host azole pressure. Similarly, the sequential isolation of TR_34_/L98H and TR_34_^3^/L98H from a single patient revealed near-identical STR profiles, differing at only one locus [55]. Together, these observations provide compelling evidence that TR expansions can arise during clinical treatment, although re-infection with environmentally evolved strains cannot be excluded. Consequently, environmental selection remains the dominant driver, as spatiotemporal analyses consistently point to a Western European origin for TR genotypes, with little evidence supporting patient-to-patient or patient-to-environment transmission [56].

**Table 1:** Known mutations in Cyp51A causing reduced susceptibility to medical azoles in *A. fumigatus*. MIC values are reported in µg/mL for ITC, VRC, POS and ISA and are taken from their first original reports or a later-reported broad cohort. MIC ranges are indicated with isolate recovery location, either clinical (CLN), environmental (ENV) or acquired by transformation (TRF). Isolate origin indicates places (multiple studies) where the *cyp51A* genotype has been found. If an isolate has never been found naturally, it was only obtained by TRF. ‘Isolates’ indicates the number of isolates used to determine the MIC, derived from the study MIC cited.

*cyp51A* Genotype	Isolate Origin	ITC MIC	VRC MIC	POS MIC	ISA MIC	First Reported	Isolates	MIC Cited
WT	CLN + ENV	<1	<1	<0.13	<1	–	–	EUCAST
TR_34_/L98H	CLN + ENV	8–>16	1–16	0.13–4	2–>16	2007 [57]	1338	CLN [40]
TR_34_^3^/L98H	CLN	>16	4–8	1	–	2020 [55]	3	CLN [55]
TR_34_/R65K/L98H	CLN	>16	8	4	4	2020 [58]	1	CLN [58]
TR_34_/L98H/F495I	CLN	>16	2	0.5	–	2018 [59]	1	TRF [59]
TR_34_/L98H/S297T/F495I	CLN + ENV	>8	4–8	0.5–1	>8	2007 [45]	47	CLN [40]
TR_34_/L98H/T289A/I364V/G448S	CLN	0.5–>16	>16	1–2	>16	2022 [60]	10	CLN [40]
TR_34_	TRF	0.5	1	0.13–0.25	–	2007 [45]	3	TRF [45]
L98H	TRF	0.5	1–2	0.13	–	2007 [45]	2	TRF [45]
S297T	TRF	0.5–1.0	1	0.25	–	2018 [59]	1	TRF [59]
F495I	TRF	0.5	0.5–2	0.03–0.13	–	2018 [59]	1	TRF [59]
TR_46_/Y121F/T289A	CLN + ENV	0.5–>16	8–>16	0.13–2	8–>16	2013 [44]	332	CLN [40]
TR_46_/Y121F/M172I/T289A/G448S	CLN + ENV	0.5–>16	>16	0.25–2	>16	2017 [50]	24	CLN [40]
TR_46_^3^/Y121F/M172I/T289A/G448S	CLN + ENV	0.5–>16	>16	0.25–2	>16	2017 [50]	17	CLN [40]
TR_46_/Y121F/T289A/S363P/I364V/G448S	CLN	1–>16	>16	0.5–2	>16	2021 [61]	78	CLN [40]
TR_46_	TRF	0.5	1	0.13	–	2015 [47]	1	TRF [47]
Y121F	CLN	0.5	2–4	0.13	–	2014 [48]	1	CLN [47]
T289A	TRF	0.13–0.25	0.5	0.13	–	2015 [47]	1	TRF [47]
TR_46_/Y121F	TRF	>8	>8	0.25–0.5	–	2015 [47]	1	TRF [47]
TR_46_/T289A	TRF	0.5	2	0.25	–	2015 [47]	1	TRF [47]
Y121F/T289A	TRF	0.13	8	0.13	–	2015 [47]	1	TRF [47]
TR_46_/F495I	ENV	>8	4	1	–	2024 [62]	1	ENV [62]
TR_53_	CLN + ENV	>8	2–4	0.5–1	8	2008 [24]	3	CLN [63]
TR_120_/F46Y/M172V/E427K	CLN	16–>16	4	0.5	–	2019 [54]	2	CLN [54]
G54A	ENV	>32	0.13	1	–	2015 [64]	2	ENV [64]
G54E	CLN + ENV	>16	0.25	1–4	0.13–0.25	2003 [30]	22	CLN [65]
G54K	CLN	>16	–	–	–	2003 [66]	2	TRF [67]
G54R	CLN + ENV	>16	0.25–4	2–>16	0.13–4	2003 [68]	3	CLN [69]
G54V	CLN	>8	0.5	1	–	2003 [68]	1	CLN [16]
G54W	CLN	>16	0.13–0.25	>16	0.13	2003 [68]	1	CLN [69]
M220I	CLN + ENV	>16	0.5	1	1–2	2005 [70]	2	CLN [69]
M220K	CLN	4–>16	1–2	1–4	1–2	2004 [31]	9	CLN [41]
M220L	CLN	>8	0.5	>8	-	2014 [71]	1	CLN [71]
M220R	CLN	>16	2	2	–	2008 [24]	1	CLN [24]
M220T	CLN	>8	0.5–4	0.25–1	–	2004 [31]	6	CLN [16]
M220V	CLN	>8	1	0.5–1	–	2004 [31]	4	CLN [31]
M220W	CLN	–	–	–	–	2010 [72]	–	N.A.
G448S	CLN	>16	0.5–>16	1–4	0.25–>16	2003 [32]	4	CLN [69]
G138C	CLN	>16	4–>8	1– >16	–	2006 [73]	10	CLN [16]
G138R	CLN	–	–	–	–	2003 [32]	–	N.A.
G138S	CLN	>16	16	2	>16	2016 [69]	1	CLN [69]
P216L	CLN	>16	1	1	–	2009 [16]	1	CLN [74]
F219C	CLN	>32	0.25	0.13	–	2013 [75]	1	CLN [75]
F219I	CLN	>16	1–8	0.5–>16	–	2012 [74]	6	CLN [74]
F219L	CLN	>4	0.5	1	2	2016 [76]	1	CLN [41]
F219S	CLN	>16	2	2	2	2016 [69]	1	CLN [69]
Y431C	CLN	>8	4	1	–	2009 [16]	1	CLN [16]
Y431S	CLN	>16	4	0.5	–	2015 [77]	1	CLN [77]
G432S	CLN	>16	2–4	0.5–4	4–16	2011 [78]	3	CLN [41]
G434C	CLN	>8	4	1	–	2009 [16]	1	CLN [16]
F46Y/M172V/N248T/D255E/E427K	CLN	0.25–2	0.5–2	0.06–0.5	–	2009 [16]	4	CLN [79]
F46Y/M172V/E427K	CLN	0.25–1	0.25–2	0.06–0.25	–	2008 [80]	11	CLN [79]

**Figure 1:**
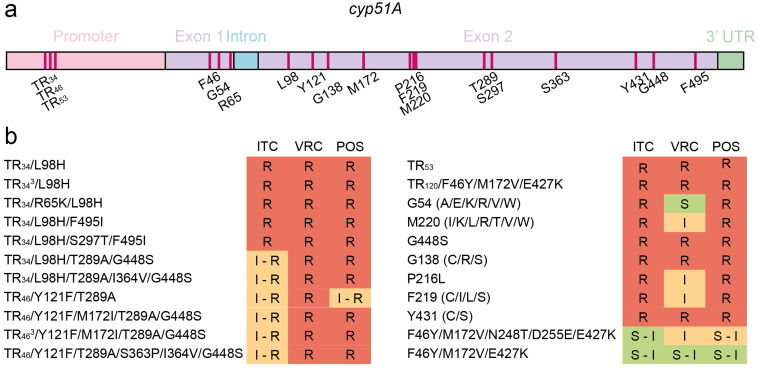
Mutations in *cyp51A* related to resistance to triazoles in *A. fumigatus*. (**a**) Amino acid hotspots for mutations in the *cyp51A* gene associated with azole resistance. (**b**) Effects of TR expansions and nonsynonymous mutations in Cyp51A on ITC, VRC and POS susceptibility as inferred from MIC values presented in Table 1. ITC and POS have similar long side arms, whereas VRC has a short side arm. While genotype–phenotype associations reflect common resistance patterns, correlation between specific mutations and susceptibility is complex and may be influenced by additional factors.

Beyond promotor TR expansions, multiple amino acid substitutions in Cyp51A, including mutations at codons G54, G138, M220 and G448, have been robustly associated with azole resistance in *A. fumigatus* (Figure 1). Substitutions at G54 (A/E/K/R/V/W) typically confer cross-resistance to ITC and POS while retaining susceptibility to VRC (Table 1) [30,66,68]. Mutations at M220 (I/K/L/R/T/V/W) similarly drive resistance to ITC and POS, with VRC MICs generally elevated relative to wildtype and G54 mutant strains [24,31,70,72]. In contrast, substitutions at G138 (C/S/R) are consistently linked to pan-azole resistance [32,69,73]. Structurally, G54, G138 and M220 cluster near the entrance to one of the two ligand access channels of Cyp51A, suggesting that substitutions at these positions selectively hinder azole entry to the heme-binding pocket [81]. Importantly, not all substitutions at these hotspots are functionally equivalent, as exemplified by G138D, which failed to alter azole susceptibility following clonal transformation [82].

Additional substitutions, including G448S, F219 (S/I/C/L) and P216L, are also recurrently observed in azole-resistant *A. fumigatus* isolates (Table 1) [32,69,74,75,76]. All are associated with ITC resistance, although their impact on other triazoles are more variable. G448S, located adjacent to the heme group, has been linked to pan-triazole resistance in some isolates, while others display intermediate MICs of 0.5 and 0.25 µg/mL for VRC and ISA, respectively [69]. The G448S substitution is thought to impair azole–heme coordination, thereby weakening drug binding [83]. Y431 is also located near the heme group, and substitutions at this location reduce the binding affinities of azoles to Cyp51A, possibly explaining the pan-azole resistance phenotype [84]. Mutations P216L and F219I have been associated with resistance to both ITC and VRC; however, for F219I, substantial phenotypic heterogeneity persists. Lastly, isolates with the combinations F46Y/M172/N248T/D255E/E427K and F46Y/M172/E427K generally show elevated MICs, with some isolates being susceptible [79], underscoring the complexity of genotype–phenotype relationships in azole resistance [74]. These observations further highlight that epistatic interactions between mutations can contribute to variability, complicating direct genotype–phenotype predictions.

These observations have direct therapeutic implications, as the activity of azoles against resistant *A. fumigatus* isolates depends not only on the underlying resistance mechanism, but also on the relationship between achievable drug exposure and the MIC. Elevated MICs for the azole used for treatment are associated with an increased likelihood of therapeutic failure, reflecting a reduced probability of achieving effective drug concentrations at the site of infection [16]. In contrast, for isolates with low-level resistance (e.g., MICs around 0.25–0.5 mg/L for POS), increasing azole exposure may partially restore antifungal efficacy. As demonstrated by Schauwvlieghe et al., high-dose posaconazole can be considered in selected cases, provided that adequate drug levels (>3 mg/L) are achieved and carefully monitored [85]. Such strategies may enable the treatment of isolates with MICs near clinical breakpoints, whereas isolates with higher MICs are unlikely to respond despite dose escalation. These findings emphasize that MIC values should be interpreted within a pharmacokinetic–pharmacodynamic framework and support individualized treatment approaches based on both susceptibility data and achievable drug exposure.

## 3. Genotype–Phenotype Variability

*cyp51A* genotypes are frequently treated as proxies for distinct azole susceptibility profiles. In broad terms, isolates harboring TR_34_/L98H are considered pan-triazole-resistant, whereas TR_46_/Y121F/T289A is primarily associated with resistance to VRC and ISA, with variable susceptibility to ITC and POS. Substitutions at G54 typically confer resistance to ITC and POS while preserving VRC susceptibility, whereas M220 mutations follow a similar pattern but often exhibit modestly elevated VRC MICs approaching the resistance breakpoint. Finally, mutations at G138 and G448 are widely considered to drive pan-triazole resistance. While these associations provide a useful heuristic framework, they also raise a critical question: how reliably does a given *cyp51A* genotype predict the resistance phenotype of an individual *A. fumigatus* isolate?

This question is sharply illustrated by a large Dutch surveillance cohort of 1979 phenotypically azole-resistant *A. fumigatus* isolates, of which 1338 carried TR_34_/L98H and 332 harbored TR_46_/Y121F/T289A without additional *cyp51A* mutations [40]. Despite their canonical resistance profiles, only 1173 (87.7%) of TR_34_/L98H isolates and 226 (80.1%) of TR_46_/Y121F/T289A sensu stricto isolates were resistant to POS, revealing substantial genotype–phenotype discordance for this agent. Even more strikingly, ITC susceptibility testing of TR_46_/Y121F/T289A isolates revealed a clear 1:1 bimodal distribution, as one population displayed high-level resistance, with MICs exceeding 16 µg/mL, whereas the second clustered around a median MIC of approximately 1 µg/mL, near the clinical resistance breakpoint [40]. It remains unclear what drives these differences in ITC activity. It is possible that the TR_46_/Y121F/T289A mutation in *cyp51A* causes ITC susceptibility of 0.5–2 µg/mL, while a commonly associated unknown mechanism with this genotype causes MICs exceeding 16 µg/mL. Alternatively, ITC phenotype variation might be due to technical issues, as ITC is highly hydrophobic and is challenging to dissolve in RPMI-1640 medium, which might affect ITC MIC readouts.

Overall, 108 of the 1670 isolates (6.5%) carrying either TR_34_/L98H or TR_46_/Y121F/T289A exhibited a phenotype falling outside the defined core MIC range for their genotype (with no core ITC MIC defined for TR_46_/Y121F/T289A) [40]. When the analysis was expanded to include all tandem repeat-containing isolates, including those with additional *cyp51A* substitutions, genotype–phenotype discordance increased substantially: 325 of 1887 isolates (17.2%) deviated from their predicted susceptibility profiles, with this fraction rising over time [40]. Isolates with additional background mutations, such as TR_34_/L98H/T289A/I364V/G448S, have greater susceptibility to ITC than TR_34_/L98H, possibly because these additional mutations come at a fitness cost (Figure 1b) [40]. This trend suggests that composite genotypes such as TR_34_/L98H/S297T/F495I and TR_46_/V120A/Y121F/M172I/T289A/G448S progressively undermine the reliability of canonical resistance categories, reflecting an increasingly complex mutational landscape in which incremental modifications to Cyp51A erode the predictive power of single-genotype assignments.

Genotype–phenotype variability is not confined to TR expansion genotypes but is equally evident among *cyp51A* point mutations. Substitutions at G54 and M220, while classically linked to resistance against ITC and POS, display notable quantitative variability in MICs, particularly for VRC, where values may range from wildtype-like to near or above the clinical breakpoint (Table 1) [16,24,69]. Similarly, mutations at G138 and G448 are frequently categorized as pan-triazole resistance determinants, yet G448S has VRC MICs ranging from 0.5 to 16 µg/mL [69]. Additional substitutions, such as F219 and P216L, further exemplify this complexity: although consistently associated with ITC resistance, their impacts on VRC and ISA remain heterogeneous, with both susceptible and resistant phenotypes reported for the same amino acid change [69,74,75].

Another example of the ambiguous Cyp51A genotype–phenotype relationship is observed in isolates carrying compound *cyp51A* substitutions such as F46Y/M172/N248T/D255E/E427K or F46Y/M172/E427K [79,80,86]. Although these genotypes are associated with elevated azole MICs, most substituted residues, except F46Y, are non-conserved and located on the exterior of the Cyp51A protein, raising questions about their direct functional relevance. Moreover, F46Y alone does not appear sufficient to confer elevated MICs [79]. Consistent with this, the transformation of *A. fumigatus* with wildtype *cyp51A* replaced by F46Y/M172/N248T/D255E/E427K resulted in substantially lower MICs than those observed in clinical isolates harboring the same genotype [79]. Together, these observations indicate that resistance in such isolates cannot be explained solely by altered azole–Cyp51A interactions. Instead, they point toward additional resistance determinants that operate independently or in combination with the *cyp51A* sequence or expression. As such, these genotypes exemplify the limitations of *cyp51A*-centric interpretations of azole resistance and underscore the need to consider broader, multilayered mechanisms that collectively shape the resistance phenotype.

## 4. Non-Cyp51A Genes Causing Triazole Resistance

A small fraction—but potentially up to 50%—of triazole-resistant *A. fumigatus* isolates display resistance phenotypes that cannot be explained by alterations in Cyp51A alone [41,72,87,88]. Given a pangenome of almost 9000 core genes and potentially 15,000 predicted genes, *A. fumigatus* possesses a vast genetic repertoire capable of modulating triazole susceptibility through mechanisms that extend beyond direct target modification [22,89,90]. Resistance-conferring mutations can arise under azole selection pressure through multiple reproductive strategies, including asexual, sexual and parasexual processes [21,22,29,91]. The species’ capacity for frequent genetic recombination, particularly during sexual reproduction, facilitates the rapid emergence and diversification of multidrug-resistant genotypes. This process effectively weakens genetic linkages, causing the rapid breakdown of associations between resistance determinants and molecular markers over short genomic distances [92,93,94]. In parallel, ongoing sporulation in environmental niches and within chronically ill patients provides continuous opportunities for clonal expansion and adaptation under drug pressure.

Importantly, azole exposure not only selects for resistance but may actively accelerate its evolution. Recent studies have identified mutations in the DNA mismatch repair gene *msh6* in a significant subset of TR_34_/L98H isolates, conferring a hypermutator phenotype characterized by mutation rates up to fivefold higher than in the wildtype [93]. Such genetic backgrounds are poised to accumulate secondary mutations that modify antifungal susceptibility, providing a plausible evolutionary framework for the emergence of resistance mechanisms that operate independently of *cyp51A* and contribute to the observed genotype–phenotype variability.

**Figure 2:**
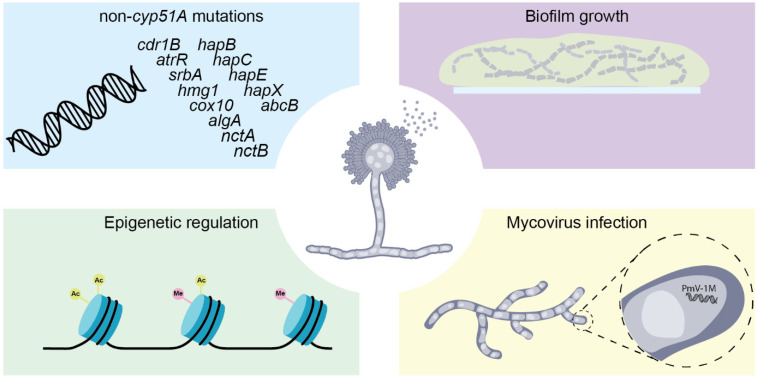
Factors contributing to genotype–phenotype heterogeneity in *A. fumigatus*, including non-Cyp51A mutations, biofilm growth, epigenetic regulation and mycovirus (PmV-1M) infection. *cdr1B* encodes for an ABC transporter; *atrR* and *srbA* encode transcription factors; *hapB*, *hapC*, *hapE* and *hapX* encode subunits of the CBC-hapX complex; *hmg1* encodes hydroxymethylglutaryl-CoA reductase; *cox10* encodes a farnesyltransferase; and *algA* encodes for a calcium signaling pathway component in *A. fumigatus*.

Besides mutations in Cyp51A, triazole resistance in *A. fumigatus* can arise through a diverse set of additional genetic mechanisms (Figure 2). In a cohort of 64 azole-resistant clinical isolates from Manchester, 43% showed no Cyp51A mutations, underscoring the clinical relevance of non-*cyp51A* resistance pathways [72]. These mechanisms may coexist with TR-based genotypes, contributing to variable susceptibility profiles, such as the inconsistent ITC resistance observed among TR_46_/Y121F/T289A isolates [40].

One of the most prominent non-*cyp51A* resistance mechanisms is the upregulation of multidrug efflux pumps, which has been linked particularly to ITC resistance [66]. *A. fumigatus* encodes a large repertoire of transporters, including 278 predicted members of the major facilitator superfamily (MFS) and 49 ATP-binding cassette (ABC) transporters [95]. Among these, the ABC transporter Cdr1B has emerged as a central determinant of clinical resistance. Its expression was elevated more than fivefold in 8 of 10 azole-resistant isolates lacking *cyp51A* mutations, and the deletion of *cdr1B* restored ITC susceptibility [96]. Conversely, *cdr1B* deletion in wildtype isolates increased azole sensitivity approximately fourfold, while *cdr1B* expression was strongly induced upon voriconazole exposure, confirming a direct and functional role in resistance [96]. Paul et al. constructed deletion alleles of two *A. fumigatus* Pdr5-like ABC transporter genes (abcA, abcB) in three genetic backgrounds and showed that the loss of *abcB* consistently increased azole susceptibility, while *abcA* phenotypes were strain-dependent [97]. An older study by Slaven et al. cloned and characterized the ABC transporter gene *atrF*, showing that its expression was fivefold higher in ITC-resistant isolates when grown in the presence of ITC [98]. Collectively, these findings establish efflux-mediated drug export as a major contributor to genotype–phenotype variability, capable of shifting MICs across clinical breakpoints independently of the Cyp51A sequence.

Efflux activity and ergosterol biosynthesis are tightly controlled at the transcriptional level, placing regulatory proteins at the core of non-*cyp51A* resistance. The fungal-specific Zn2-Cys6-type transcription factor AtrR plays a pivotal role in coordinating resistance responses by directly regulating both *cyp51A* and *cdr1B* expression [99]. AtrR binds to the promotor regions of approximately 900 genes, including the TR_34_ and TR_46_ regions of *cyp51A* and the promotor of *cdr1B* [51]. Gain- or loss-of-function alterations in AtrR profoundly modulate azole susceptibility, as the deletion of the *atrR* gene in an azole-resistant Cyp51A G54E *A. fumigatus* isolate resulted in hypersensitivity to azoles [99]. Similarly, the sterol-regulatory element-binding protein SrbA, a highly conserved transcription factor, governs ergosterol biosynthesis under hypoxic and stress conditions and is required for full azole resistance and virulence [100]. Altered SrbA activity reshapes sterol homeostasis and drug susceptibility without necessitating structural changes to Cyp51A [101]. Another important regulatory component is HapE, a subunit of the CCAAT-binding transcription factor complex that specifically recognizes the regulatory CCAAT sequence in the promotor regions of numerous eukaryotic genes [102]. Whole-genome sequencing (WGS) and sexual crossing of resistant isolates without *cyp51A* mutations revealed a P88L substitution in HapE that could confer azole resistance [103]. Mutations in *hapE* disrupt the transcriptional repression of ergosterol biosynthesis genes, resulting in elevated azole MICs even in the absence of *cyp51A* coding mutations. HapE, together with HapB and HapC and two copies of HapX, forms the CBC-HapX complex, which regulates genes involved in ergosterol biosynthesis [52,104]. Disruption of this complex—for example, through the deletion of *hapB*—significantly prolongs the nuclear retention of *SrbA*, ultimately increasing *Cyp51A* expression and reducing azole susceptibility [105]. In addition to the CBC-HapX complex, the transcriptional regulators negative cofactor two A and B (NctA and NctB, respectively) also play key roles in controlling the ergosterol biosynthesis pathway. Loss of these proteins similarly confers azole resistance, notably without compromising virulence [106]. Together, these transcription factors exemplify how regulatory rewiring can generate resistance phenotypes that diverge from genotype-based expectations.

Additional resistance determinants act upstream of Cyp51A within the ergosterol biosynthesis pathway. Rybak et al. demonstrated that the replacement of mutant *cyp51A* alleles with a wildtype *cyp51A* gene restored susceptibility in only one of ten triazole-resistant clinical isolates, providing strong evidence for alternative resistance mechanisms [88]. In a broader collection of 21 resistant isolates, 11 carried mutations in *hmg1*, encoding hydroxymethylglutaryl (HMG)-CoA reductase, which catalyzes the first committed step in ergosterol biosynthesis. Missense mutations within the sterol-sensing domain of Hmg1 increased triazole MICs four- to eightfold, whereas restoration of the wildtype *hmg1* allele in a pan-triazole-resistant isolate harboring a deletion of F262 fully restored susceptibility [88]. The contribution of *hmg1* mutations to triazole resistance has since been independently validated [107,108,109,110]. Interestingly, a *cyp51B hmg1* combination mutant showed a pan-azole-resistant phenotype, with higher levels of expression of Cyp51A, Hmg1 and Cyp51B during VRC exposure [111]. These findings position *hmg1* as a critical modifier of azole susceptibility, capable of reshaping sterol flux and buffering the inhibitory effects of azoles downstream.

Other genes have been implicated in non-*cyp51A*-mediated resistance, often through effects on drug uptake or cellular stress adaptation. A single amino acid substitution (R243Q) in Cox10, a farnesyltransferase involved in mitochondrial function, was shown to reduce itraconazole uptake [76]. Additionally, the deletion of *algA*, encoding a calcium-dependent protein, increased the frequency of non-*cyp51A*-mediated azole resistance, suggesting a role in stress tolerance or cellular homeostasis [76]. Importantly, the roles of all these genes have been functionally validated through targeted transformation experiments, providing compelling evidence for their causal involvement in azole resistance independently of Cyp51A.

## 5. Non-Somatic Azole Resistance

Beyond heritable genetic variation, azole resistance in *A. fumigatus* is profoundly shaped by various environmental and physiological factors, including biofilm growth, epigenetic regulation and mycovirus infection (Figure 2). Growth conditions can directly modulate antifungal susceptibility without altering the underlying genotype [112]. For example, alternative nutrient sources such as glucosamine and *N*-acetylglucosamine remodel the cell wall architecture and alter susceptibility to echinocandins [113]. Similarly, in vivo hyphal growth within biofilms generates extracellular matrices that restrict antifungal penetration [114,115,116] and establishes oxygen gradients promoting hypoxic microenvironments [117]. These features significantly elevate apparent drug resistance; for example, Seidler et al. demonstrated that triazole MICs in *A. fumigatus* biofilms can be up to tenfold higher than those observed in planktonic cultures [118]. Biofilm maturation is associated with phase-dependent increases in azole tolerance, partly driven by enhanced efflux pump activity. As shown by Rajendran et al., more mature hyphal stages exhibit markedly increased VRC resistance alongside the upregulation of efflux transporters such as *AfuMDR4*, an effect that is further induced upon azole exposure [119].

Environmental adaptation can also contribute to stable, non-*cyp51A*-mediated azole resistance phenotypes. The experimental evolution of strain AF293 under hypoxic and high-glucose conditions generated the EVOL20 strain, which exhibited an H-MORPH biofilm phenotype linked to a mutation in *hrmA* [120]. This adaptation was associated with altered virulence and increased *Cyp51A* expression, suggesting that environmental conditioning can indirectly influence azole susceptibility. Notably, similar morphologies have been observed in clinical isolates, although their impacts on treatment outcomes remain unclear.

Epigenetic regulation introduces an additional, reversible layer of resistance modulation. Histone modifications, including acetylation and methylation, influence fungal stress responses and antifungal tolerance without altering DNA sequences [121]. The acetylation of lysine K27 in Hsp90, for instance, reduces tolerance to both caspofungin and voriconazole [122], while the deletion of histone deacetylases (HDACs) has been associated with altered virulence, morphogenesis and antifungal susceptibility [123,124]. Such epigenetic mechanisms enable rapid, condition-dependent phenotypic plasticity, allowing *A. fumigatus* to transiently withstand antifungal pressure in fluctuating host and environmental niches.

Finally, extra-chromosomal elements such as mycoviruses may further modulate antifungal susceptibility. Mycoviruses are present in a subset of *A. fumigatus* isolates and can alter fungal physiology and stress responses [125,126]. While their impact on azole resistance in *A. fumigatus* remains unresolved, studies in other fungi, such as *Penicillium digitatum*, show that mycovirus infection can reduce triazole resistance [127]. These observations suggest that virus–fungus interactions may influence antifungal susceptibility and could contribute to the unexplained variability in azole responses [128,129].

## 6. Diagnostics for Azole Resistance

The accurate detection of azole resistance in *Aspergillus fumigatus* is essential in guiding antifungal therapy and improving patient outcomes. This is particularly critical in IA, where delays in initiating effective treatment are strongly associated with increased mortality [130]. Moreover, resistance to azoles was associated with 20–25% excess overall mortality at day 90 in patients with IA [131,132,133]. As azole-resistant variants continue to emerge worldwide, diagnostic strategies must support both timely clinical decision-making and reliable epidemiological surveillance.

**Figure 3:**
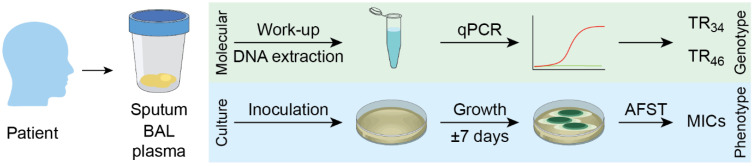
Regularly used diagnostic pathways for azole resistance detection in *Aspergillus*, showing clinical sample processing via molecular-based methods (qPCR) and culture, followed by antifungal susceptibility testing (AFST).

Following the acquisition of a clinical specimen, such as sputum, bronchoalveolar lavage (BAL) or plasma, *A. fumigatus* can be detected by culture-based or molecular methods (Figure 3). Culture positivity rates vary depending on the methods of culturing, ranging from 0% to 16% for conventional sputum cultures and 37% to 54% in high-volume sputum cultures [134,135,136,137], whereas those for bronchoalveolar lavage samples range from 10% to 60% for conventional cultures and 20% to 100% by high-volume culture [134,138]. When cultures are positive, azole resistance can be assessed phenotypically using screening assays such as VIPcheck^TM^ or by standardized broth microdilution methods to determine minimum inhibitory concentrations (MICs) (Table 2) [139]. These approaches directly measure fungal growth in the presence of antifungal agents and remain the reference standard for assessing susceptibility across a broad range of compounds.

A major advantage of phenotypic testing is its ability to capture the cumulative effects of all resistance mechanisms present in an isolate, including those not yet genetically characterized. Furthermore, multiple colonies (if present) can be tested with screening agar, as mixed-genotype infections are common [40]. However, culture-based diagnostics can lack sensitivity and are inherently slow, requiring several days for fungal growth and subsequent MIC determination, potentially minimizing the clinical utility of testing. This delay is clinically problematic in IA, where early initiation of appropriate therapy was found to be critical for survival [131]. Moreover, approximately up to 80% of IA cases are culture-negative, severely limiting the applicability of phenotypic testing in routine diagnostics [41,140,141].

To overcome the low sensitivity and slow turnaround time of culture, molecular assays have been developed to detect azole resistance-associated mutations directly from clinical material [142]. PCR-based platforms, including real-time PCR and melting curve analysis, enable the rapid identification of the most prevalent *cyp51A* resistance genotypes, such as TR_34_/L98H and TR_46_/Y121F/T289A (Table 2). These assays bypass the need for fungal growth and substantially reduce the time to results, facilitating earlier therapeutic adjustments.

Real-time qPCR applied directly to clinical samples generally has a higher detection rate than culture-based methods [136]. However, resistance PCRs target *cyp51A*, a single-copy gene, resulting in markedly reduced sensitivity compared with species-specific *Aspergillus* PCR assays that amplify multicopy regions—most commonly, short amplicons within the rRNA operon [142]. This limitation becomes particularly relevant in mixed infections, where resistant and susceptible strains may coexist. In such cases, resistant subpopulations can fall below the detection threshold, leading to false-negative results and potentially inappropriate antifungal therapy.

Despite their speed, current molecular diagnostics face important conceptual limitations [28]. Commercial assays primarily focus on the most common *cyp51A* alterations, while an expanding range of resistance mechanisms, including non-*cyp51A* mutations, efflux pump upregulation, transcriptional rewiring and cryptic species with intrinsic resistance, remain undetected [130]. Additionally, widely used resistance PCR platforms such as AsperGenius^®^, MycoGENIE^®^ and Fungiplex^®^ do not interrogate the full *cyp51A* coding region but instead target specific drug resistance-associated mutation hotspots [27,28]. Assuming 100% analytical sensitivity, a retrospective analysis of *cyp51A* mutations identified in 1979 azole-resistant *A. fumigatus* isolates from the Dutch collection [40] indicated that AsperGenius^®^ would have detected 94.9% of resistant isolates, Fungiplex^®^ 94.8% and MycoGENIE^®^ 72.4%. These figures illustrate the high diagnostic value of targeted molecular assays in settings where TR-associated *cyp51A* mutations dominate, as is currently the case in the Netherlands. However, in regions where resistance is more genetically diverse, or if clinically driven, hotspot-based assays are expected to miss a substantially larger fraction of resistant isolates [72]. For example, in Denmark, the prevalence of non-Cyp51A-mediated azole resistance was reported to be 40% (27/66 clinical isolates) [41]. A recently developed pyrosequencing assay was able to capture nearly the whole *cyp51A* gene and was validated on 326 clinical specimens, where it was substantially more sensitive than culture [143]. Out of the 326 *Aspergillus* qPCR-positive respiratory specimens, 275 (84.4%) were successfully sequenced, while 94 (28.8%) were cultured by high-volume culture. Despite the low culture positivity, 21 pan-azole-resistant *A. fumigatus* isolates were detected by high-volume culture, while pyrosequencing identified no mutations in *cyp51A* or was negative, highlighting the complex genotype–phenotype relationship [143]. The sensitivity of *Aspergillus* PCRs strongly depends on the type of clinical material, as the detection of resistance markers in blood is often unsuccessful due to low fungal DNA loads [142]. Although melting curve-based assays such as AsperGenius^®^ can sometimes identify mixed infections, the overall genotype–phenotype relationship remains difficult to capture using targeted molecular methods alone [144].

## 7. Implications and Future Directions for Diagnostics

Together, these observations underscore the complexity of the genotype–phenotype relationship underlying azole resistance in *A. fumigatus*. While molecular assays enable the rapid and sensitive detection of common resistance markers, they incompletely represent the diversity of resistance mechanisms encountered in clinical isolates. This limitation is particularly relevant in patients undergoing prolonged azole therapy, where selective pressure promotes the emergence of rare or novel resistance mutations. Mixed infections further complicate interpretation, as multiple genotypes and phenotypes can coexist within a single host, leading to misleading diagnostic outcomes [40].

At present, genotype-only approaches are therefore insufficient for fully reliable clinical decision-making. Although the detection of TR_34_/L98H or TR_46_/Y121F/T289A does not allow accurate prediction of the exact resistance phenotype, especially considering emerging variants, a pragmatic clinical strategy is to avoid azole therapy altogether once a resistance marker is detected—noting that alternatives to azoles, such as liposomal amphotericin B treatment, have possible detrimental side effects [145]. If the prevalence of azole resistance is over 10%, first-line therapy with VRC in combination with echinocandin or liposomal amphotericin B is recommended, awaiting antifungal susceptibility testing, as is the case in the Netherlands [35]. This approach prioritizes patient safety but also highlights the diagnostic gap between rapid genotyping and comprehensive phenotypic assessment. In the absence of one of these genotypes being detected, azole resistance cannot be excluded given the diverse genetic resistance mechanisms listed above that are not targeted by these tests, as well as the fact that these tests are not 100% sensitive for the detection of these markers.

Next-generation sequencing (NGS) offers a powerful framework to bridge this gap. Whole-genome sequencing (WGS) can identify both established and novel resistance mutations, detect cryptic species and capture mixed infections that may escape PCR-based diagnostics [146]. Importantly, sequencing is not restricted to *cyp51A* and enables the simultaneous interrogation of efflux transporters, transcription factors, stress response pathways and other loci implicated in antifungal tolerance [147]. Pan-genome analyses based on de novo assemblies further extend this approach by revealing the full genetic repertoire of the species, rather than relying on a single reference genome [90]. The principal challenge of NGS-based diagnostics lies in interpretation, given that many genetic variants lack clear functional annotation unless linked to phenotypic susceptibility data.

## 8. Conclusions

Advancements in genotype–phenotype diagnostics have vastly improved patient outcomes. However, with emerging cryptic species, mixed infections and co-infections, the need to continually develop and optimize molecular detection strategies increases. The future of azole resistance diagnostics will depend on systematic genotype–phenotype integration, combining broad genomic profiling with standardized susceptibility testing and functional validation. Such efforts could lead to curated resistance-associated variant panels that extend beyond Cyp51A, incorporate emerging mechanisms and are continuously updated as new data become available. Ultimately, embedding NGS within diagnostic workflows and explicitly linking genetic variation to phenotypic resistance will enable diagnostics that are not only rapid and sensitive but also mechanistically informative and clinically actionable.

## Figures and Tables

**Table 2:** Commercial methods of detecting azole resistance in *Aspergillus fumigatus*. Phenotypic methods allow for antifungal susceptibility testing to determine MIC values, whereas genotypic methods reveal only common azole-resistant genotypes.

Name of Test	Company	Test Description	Relevance to Resistance
**Phenotype-based**			
Sensititre YeastOne	Thermo Fisher Scientific and Trek Diagnostic systems	Colorimetric broth microdilution plate for AFST	MIC for a range of azoles
ETEST	bioMérieux	Strips with azole gradients	MIC for a range of azoles
MIC test strip	Liofilchem	Strips with azole gradients	MIC for a range of azoles
Micronaut-AM	Bruker	Colorimetric microdilution	MIC for a range of azoles
VIPcheck^TM^	Mediaproducts BV	Agar quadrants with azoles and control	Screening of growth inhibition
Neosensitabs^TM^	Rosco	Azole disk diffusion assay	Breakpoint testing for a range of azoles
**Genotype-based**			
AsperGenius^®^	PathoNostics BV	Multiplex qPCR of TR_34,_ L98H, Y121F and T289A with Tm analysis (450, 530, 598 and 645 nm) [27]	Rapid identification of TR_34_/L98H and Y121F/T289A
MycoGENIE^®^	Adamtech	Multiplex qPCR of TR_34_ and L98H [28]	Rapid identification of TR_34_/L98H
Fungiplex^®^ *Aspergillus* Azole-R	Bruker	Multiplex qPCR of TR_34_ and TR_46_ [28]	Rapid identification of TR_34_ and TR_46_

## Data Availability

No new data were created or analyzed in this study. Data sharing is not applicable to this article.
